# Assessment of soil organic carbon fractions and carbon management index under different land use types in Olesharo Catchment, Narok County, Kenya

**DOI:** 10.1186/s13021-018-0091-7

**Published:** 2018-02-12

**Authors:** Bernice M. Sainepo, Charles K. Gachene, Anne Karuma

**Affiliations:** 1 Department of Land Resource Management and Agricultural Technology, University of Nairobi, P.O Box 29053, Nairobi, 00625 Kenya; 2 Mainstreaming Sustainable land management in Agro-pastoral production, Kenya Project, United Nations Development Programme Kenya (UNDP-K), P.O. Box 30218, Nairobi, 00100 Kenya

## Abstract

**Background:**

The changes in land use and land cover have a strong effect on the total soil organic carbon, its fractions and its overall soil health. This study carried out in Olesharo Catchment, Kenya, was to quantify the differences in total organic carbon (TOC), particulate organic carbon (POC), mineral organic carbon (MOC) and carbon management index (CMI) among four land use types: grasslands, shrublands, agricultural lands and barelands. It was also purported to evaluate the use of CMI as an indicator for soil degradation or improvement in response to land use and land cover changes.

**Results:**

The results of the study show that the mean values of TOC, POC and MOC are significantly different between land use types. Thus, shrublands have significantly higher TOC (22.26 g kg^−1^) than grasslands (10.29 g kg^−1^) and bare lands (7.56 g kg^−1^). They also have significantly higher POC (7.79 g kg^−1^) and MOC (10.04 g kg^−1^) than all the other land use types. The agricultural lands have higher CMI than grasslands (53% vs 41% relative to shrublands) suggesting that grasslands face serious degradation through overgrazing.

**Conclusions:**

This study shows that different land use types have an influence on soil organic carbon pools, and consequently on the CMI, the CMI could be used as an indicator for soil degradation or improvement in response to land use and land cover changes.

## Background

Increasing anthropogenic disturbances especially, on land use/cover change (LULCC), is the major cause of soil quality deterioration in the world [1]. Soil organic carbon (SOC) has recently gained prominence in assessment of soil quality since it compoundly affects chemical, physical and biological aspects of the soil. Though described by some as the least most understood component of the soil because of its dynamism, [2] SOC has been linked to its potential role in carbon sequestration through proper management of land use and cover types [3]. Land use and cover types influence C fluxes in an ecosystem; through litter quality, deposition and turnover rate. Although SOC is an indicator of soil quality, conceptualization of soil fractions can be used to detect even slight changes in management and regulate degradation [4, 5].

Soil organic matter can be divided into several fractions depending on their densities. Labile fraction (LF) is the most prominent, partly due to its high turnover rate plus it is easily affected by management systems as well as erosion [6–8]. Labile fraction has been described in various ways by soil scientists, including particulate organic carbon (POC) (53–2000 µm), light fraction organic carbon (LFOC) (density of < 2.0 g cm^−2^), readily oxidized carbon (ROC) (easily oxidized by potassium permanganate), soil microbial biomass carbon (SMBC) and dissolved organic carbon (DOC), etc. [9–11].

The labile fraction (LF) consists of the mineral-free SOM composed of partly decomposed plant and animal residues which turn over rapidly and have a specific density that is comparatively lower than that of soil minerals [12]. Agricultural soils have been identified as having the lowest LF [13, 14] due to high disturbances by tillage practices and harvesting of crop residues. In native land cover types (forests, grasslands, shrublands) however, high LF has been recorded due to high litter input and controlled soil temperature. Grazing has been seen to increase lability of carbon through activation of microbial activity by enzymes found in the saliva and dung from herbivory especially in warm temperatures [15–18]. Moreover the removal of biomass promote plant regrowth hence expedite nutrient cycling within the rhizosphere. With increase in grazing intensity, LF has been seen to significantly reduce [11], attributable to low litter deposition, high mineralization due to exposure to surface temperature and intensive erosion.

Stable fraction (SF) accounts for 90% of the total organic carbon (TOC) in terms of particle size distribution [6]. Most studies show that SF due to its recalcitrant nature is not easily affected by land use or management practices [19], while others show that this fraction is more affected than the labile portion [20, 21]. The SF arguable is said to be resistant to management systems due protection from external factors by sorption on fine particles. Its inaccessibility to decomposing microbes is due to dominance of clay particles that strongly adsorb the carbon protecting it from enzymatic action leading to the humification process [22].

There are different techniques that partition the fractions into functional pool. In this study the physical fractionation based on particle size of organic matter was used as opposed to the conventional KMnO_4_. Researches against the latter address the limitations that the concentrations are often too strong therefore detection of changes in the lability often goes unnoticed ([23]. Moreover, other studies show that the reaction times are not standard as they differ with the soil sample moisture and the decomposition of KMnO_4_ when exposed to light [9]. Whereas in support of physical fractionation, the process is able to disintegrate the POC particles to effectively detect the LF as opposed to the chemical method which is a surface attack and may provide underestimate values of the fractions [24]. Therefore the use of sieves to separate SOC fractions was employed following the study by [25] where labile fractions are to be found between sieves of sizes 53–250 μ and the stable ones < 53 μ.

Although total soil carbon varies with soil management, it is not as sensitive as the LF in short durations [26]. Therefore, calculation of the lability of SOC within each land cover type can be used as an early indicator for soil degradation or improvement in response to different management practices. In order to use more sensitive indicators, the development of carbon management index (CMI) has been used in different land uses to evaluate the capacity of a land use to promote soil quality [4, 27]. It involves the calculation of lability which is a ratio of the labile carbon to the non-labile carbon. Studies that use CMI as an assessment tool are rare, therefore the objective of this study is to investigate the SOC dynamics in each LULUCs types of the Olesharo Catchment area, Narok, Kenya and develop a CMI.

## Methods

### Description of study site

**Fig. 1:**
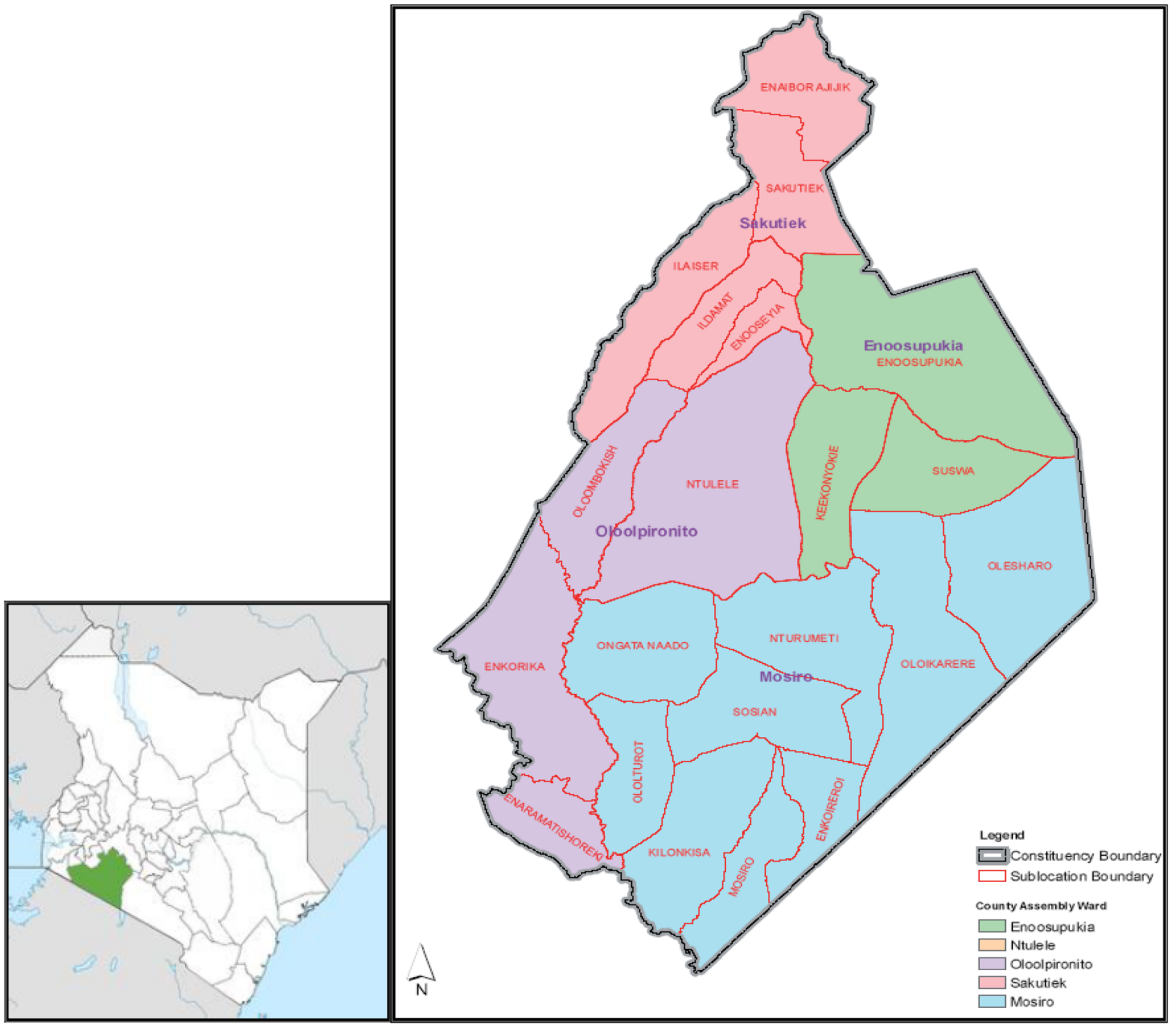
The study area in Narok County (Source: Narok District Environment Action Plan 2009–2013)

The study was carried out in Suswa Location (Fig. 1), Narok County located in the Southwest of Kenya. The County lies between longitudes 34°45′E and 36°00′E and latitudes 0°45′S and 2°00′S. The topography ranges from a plateau with altitudes ranging from 1000 to 2350 m a.s.l. at the southern parts to mountainous landscape (3098 m a.s.l) at the top of the Mau escarpment in the North [28–30].

The catchment is located within agro-climatical zones (ACZ) IV which is semi-humid to semi-arid [31]. The area experiences a bi-modal pattern of rainfall with long rains expected from mid-March to June and short rains from September to November. The local fluctuations in topography influences the rainfall distribution patterns, with the highlands receiving as high as 2000 mm year^−1^ while the lower and drier areas receiving less than 500 mm year^−1^ [32].

The Suswa area has steep gradients and volcanic-ash soils, mainly Andosols, which are prone to erosion. There are visible patches of bare land that have developed due to overgrazing. The Suswa hill is dominated by an intricate network of deep gullies reaching to 4 km in length, 25 m deep and widths of over 30 m [33]. Geomorphologically, there are pronounced cattle tracks and evidence of intense runoff and flash floods during the rains [34]. The area is dominated by scattered acacia tree species and *Thaconathusz camphoratus* which is an indication of dry weather conditions and depressed rainfall amounts [35].

### Land use

Narok County has diverse land use types spanning the agroecological zones that occur in the area. The catchment is found within the Narok County which is predominantly a semi-arid climate. Olesharo is found within the lower elevations of the County where there is a prominent transition from pastoralism to agropastrolism. The area is dominated by shrubland and grassland with patches of agricultural land and bareland (Table 1). Croplands have grown in the recent decade as a way to diversify production due to the changing climate. Farming is a monocrop of maize (Kenyan staple crop), and/or an intercrop of maize and beans. Sheep, goats and beef/dairy cattle is the predominant livelihood activity, with bee keeping in selected households [29, 36]. The area is also populated with wildlife which is exploited for tourism and ecotourism [37]. The community land has now been partitioned therefore wildlife and livestock mobility is curtailed; this in turn has had severe detrimental effects on soil erosion.

**Table 1:** Land use/cover change in Mount Suswa Catchment (1985–2011)

Land use/cover	1985 area (km^2^)	%	2000 area (km^2^)	%	2011 area (km^2^)	%	%change 1985–2000	%change 2000–2011	%change 1985–2011
Built up area	0.77	0.19	0.91	0.24	1.30	0.32	+ 18.18	+ 42.86	+ 68.83
Agricultural land	1.00	0.02	15.33	3.81	23.16	5.76	+ 1433	+ 51.08	+ 2216
Shrubland	231.1	57.4	170.6	42.4	237.8	59.1	26.18	+ 39.39	+ 2.90
Bareland	1.21	0.30	12.44	3.11	2.46	0.61	+ 928.1	+ 405.69	+ 103.3
Grassland	166.71	41.45	188.92	46.97	137.68	34.2	+ 13.32	− 27.12	− 17.41

### Suswa soils

The Suswa area has humic andosols, well drained, relatively deep, dark brown, friable and smeary, sandy clay to clay, with acidic humic topsoil [29, 38]. These soils have sand to clay ratio of 2:1 on average for the horizons studied [39]. The high silt/clay ratio, low organic matter and high bulk density which may be due to compaction as a result of continuous grazing in the area, among other factors, have made the soils more vulnerable to erosion. The soils are stratified with hard pans underlain by soft clayish strata that are readily eroded [36].

### Sampling design and soil sampling

Sites were selected to minimize soil variability. Six plots per each LUT of 30 × 30 m were randomly selected were laid on the different land use types that were identified using the Landsat maps): agriculture, bareland, grassland and shrubland. In each plot, an auger was used to collect disturbed soil samples from the centre and four corners of the plot at 0–15 and 15–30 cm depth. The samples taken from the corresponding depths were thoroughly mixed and bulked into one composite sample of about 500 g. At the centre, soil core rings (5 cm diameter) were used to collect undisturbed soil to measure soil bulk density. Geographical position and elevation of each plot were also recorded. Forty-eight soil samples per land use were collected making a total of 96 samples.

### Soil physical and chemical analysis

The SOM was fractionated following procedures described by [25]. Air-dried sub samples were sieved and 20 g placed in 250 ml plastic bottle. 70 ml of sodium hexa-metaphosphate solution was added and the mixture shaken for 15 h on an end to end shaker. The contents were passed through a series of sieves (2 mm, 250 and 53 μ) and the fractions collected dried at 50 °C for 48 h in an air oven. The 53–250 μ fraction was referred to as labile SOM. All the material that passed through the 53 μ sieve was collected in a flask, swirled to mix thoroughly and a sample of 100 ml taken and oven dried. This sample was referred to as the stable SOM. The oven-dried fractions were ground using mortar and pestle to a very fine material, sieved through a 0.149 mm sieve and analysed for SOC [40].

### ENpoc and carbon management index

The enrichment ratio of the labile carbon, was calculated by dividing it by the total organic carbon of the same land use. Carbon management index is an assessment model that shows how a particular land use affects the soil quality relative to a reference land use soil.

The index is formulated as follows:

1 $$CMI = CPI * LI * 100$$

where CPI is the carbon pool index and LI is the lability index of the soil under a particular landuse [4].

2 $${\text{CPI}} = \frac{{{\text{Total carbon in the treatment g}}\;{\text{kg}}^{ - 1} }}{{{\text{Total carbon in the reference g}}\;{\text{kg}}^{ - 1} }}$$

3 $$LI = \frac{\text{L in the treatment}}{\text{L in the reference}}$$

where L is carbon lability of the soil

4 $${\text{L}} = \frac{\text{Content of labile C}}{\text{Content on non-labile C}}$$

In this study, the native shrubland was used as reference land use. This is because it has been under rehabilitation for the last 4 years and it is enclosed from grazing and other disturbances.

### Statistical analyses

Analysis of variance (ANOVA) and Duncan’s multiple range test (DMRT) for comparison of means were performed using software SAS 9.1.3. The statistical significance was determined at P < 0.05. Effects of land use and soil depth on SOC fractions were analysed by a two-way ANOVA. A simple linear regression analysis was used to reveal the relationship between TOC and its fractions.

## Results and discussion

### Total soil organic carbon

Shrublands recorded the highest TOC with 22.26 g kg^−1^ the surface layer and 7.56 g kg^−1^ in the sub-surface layers.

**Fig. 2:**
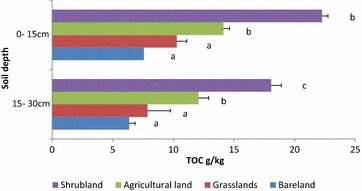
Total organic carbon (TOC) under different land cover types. Means in the same colour with different letters indicate highly significant (P < 0.05)

The TOC (Fig. 2) in SH was the highest in the surface layer (22.26 g kg^−1^) which was significantly different (P < 0.05) from the other land use types probably because it was fenced from grazing. BL had the lowest with 7.56 g kg^−1^ due to low surface cover. Total organic carbon was significantly different between all the LUTs at 0–15 cm. In the sub-surface, BL and GR were not significantly different, and both were low compared to SH and AG. Shrubland recorded the highest TOC (18.06 g kg^−1^) which was significantly different from AG (12.07 g kg^−1^). TOC was higher in the 0–15 cm than in 15–30 cm.

### Particulate organic matter

The POC was significantly highest in SH and lowest in BL across all layers.

For POC, SH had the highest (7.79 g kg^−1^) which was significantly different from the other LUTs. AG had (3.82 g kg^−1^) while GR had (2.46 g kg^−1^) which was not significantly different from BL (1.51 g kg^−1^) at the surface layer. At 15–30 cm SH and AG were significantly different at 4.93 and 2.70 g kg^−1^ respectively. In GR and BL, the POC was lower compared to the other LUTs but were not significantly different from each other at 1.37 and 1.08 g kg^−1^ respectively.

### Mineral organic matter

The MOC was higher than the POC in all the land use types.

Mineral organic carbon at the surface layer was higher than in 15–30 cm across all the LUTs. All the LUTs were significantly different in mean MOC, with SH (10.04 g kg^−1^) being the highest. AG (8.17 g kg^−1^), GR (6.49 g kg^−1^) and BL (4.24 g kg^−1^) recorded the lowest. At 15–30 cm, SH was the highest (8.15 g kg^−1^) and was significantly different from the other LUTs. AG and GR were not significantly different from each other recording (6.10 g kg^−1^) and (5.23 g kg^−1^) respectively. BL was the lowest at (3.60 g kg^−1^).

### Carbon management index

As shown in Table 2 the carbon EN_POC_ is highest in SH and lowest in BL. The CMI was highest in AG and lowest in BL. In this study, SH was taken to be the reference land use type.

**Table 2:** Effects of land use types on carbon management index at different depths

	0–15 cm				15–30 cm			
	EN_POC_%	CPI	LI	CMI	EN_POC_%	CPI	LI	CMI
BL	19.97a	0.34a	0.80a	31.00a	17.00a	0.36a	0.59a	22.77a
GR	23.95a	0.46b	0.81a	41.00ab	17.40a	0.46a	0.64ab	28.93a
AG	27.03c	0.64c	0.82a	53.00b	22.37b	0.68a	0.70c	65.73b
SH	34.99d	1d	1a	100c	27.30b	1c	1c	100c

Means with letters within are statistically different. SH is the reference land use type

*GR* grasslands, *SH* shrublands, *AG* agricultural lands, *BL* barelands, *EN*_*POC*_ enrichment ratio of POC to TOC, *CPI* carbon pool index, *LI* lability index, *CMI* carbon management index

The EN_POC_ was highest in SH (34.99%) which was significantly different from the other LUTs. Barelands recorded the lowest EN_POC_ of 19.97% followed by GR at 23.95% and AG at 27.03% at 0–15 cm depth. In the sub-surface, the EN_POC_ were lower than the surface layer. Shrubland had the highest EN_POC_ at 27.30% and which was significantly different from the others at 22.37, 17.40 and 17% for AG, GR and BL respectively. The CMI was highest in AG followed by GR then the least was BL (53, 41 and 31%) respectively in the surface layer. At 15–30 cm, the trend was similar with AG (65.73%) > GR (28.93%) > BL (22.77%) with AG being significantly different from both GR and BL.

## Discussion

### Total organic carbon (TOC)

**Fig. 3:**
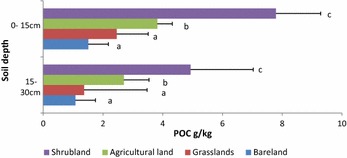
Particulate organic matter (POC) under different land cover types. Means in the same colour with different letters indicate highly significant (P < 0.05)

Shrublands had the highest TOC (Fig. 3). This is attributed to the recovery of above and below ground biomass found in the SH which is significantly higher than in AG and in GR. The litter deposition encourages turnover combined with a higher soil moisture content which is high due to the canopy provided by the trees found in this land use types. These results are similar to research done by [41] in the southern ASALs of Kenya, illustrating that SH increases TOC due to high carbon inputs. Although, other studies show that its root material has a greater influence on SOC than litter in the short term [42]. In addition, vegetation cover protects loss of SOC from the surface compared to other LUTs in the catchment. Other work on erosion studies have shown that protective cover over the surface reduces the impacts of wind and water erosion on surface horizons [7, 43–47]. Total organic carbon was lower in AG compared to SH. This may be due to the tillage practices that destroy soil aggregation and exposes organic matter to factors that encourage faster decomposition rate to carbon inputs [48], in Ethiopia showed that minimal disturbances on soil surfaces encourage microbial activity which increases TOC in the soil. Moreover, the harvesting of above ground biomass for animal feed instead of leaving it as stubble may also contribute to lower TOC [49, 50].

In the GR, the TOC was unexpectedly lower which may be attributed to the high grazing intensity within the catchment. Overgrazing affects carbon fluxes whereby the carbon inputs are less than the carbon outputs. Moreover, the cattle tracks in the GR increase the bulk density of the area therefore discouraging shoot emergence and encourage surface runoff. The area experiences high erosion rates [33, 36] which selectively carries away the SOC on the surface since it has a light density [51, 52]. A study done in Northern China on degraded grasslands showed that there was up to a 50% loss of SOC due to exposure of the surface resulting from land use change and overgrazing. This is contrary to a research done by [53] which showed that grasslands have higher capacity to store SOC than SH, however in this study area there was controlled grazing. Differences were seen down the profile as TOC was higher in 0–15 cm than in 15–30 cm. This can be attributed to higher rates of inputs of litter in the surface compared to roots in the sub-surface. Furthermore there is minimal rainfall in the area which discourages movement of carbon to the lower horizons [54].

The lower TOC in GR compared to SH can be attributed to the distribution of plant root systems which [55] suggest has more influence on soil organic matter than climate. The plant function types influence the vertical distribution of SOC within the profile [56] where grasses have a shallow root profile while shrubs have a deeper root profile. This can explain the higher TOC in SH and lower in GR in the sub-surface horizon. The presence of shrub roots in the lower horizons increases the TOC concentration with root exudates, microbial soil biomass and dehydrogenase activity [57, 58].

### Particulate organic carbon (POC)

**Fig. 4:**
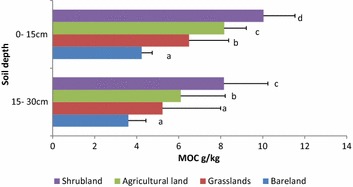
Mineral organic carbon (MOC) under different land cover types. Means in the same colour with different letters indicate highly significant (P < 0.05)

For the soil fractions studied, POC was the fraction most affected by land use within the catchment (Fig. 4). In SH, POC was the highest which can be attributed to higher litter deposits which have higher labile carbon [59, 60] that encourages microbial vitality and quantity. The SH are fenced, which regulate grazing and disturbance by both livestock and wildlife, moreover, the area has several physical soil management structures to reduce soil erosion and this may have contributed to higher POC. Similar results were found in in the central Himalayan region by [61] who showed that land use types that were undisturbed had higher POC due to accumulation of carbon that are protected by soil aggregates.

Agricultural land had lower POC than SH (Fig. 4). This is attributable to labile carbon that is highly dependent on management practices. Cultivation for example breaks down protective macroaggregates that expose the POC to higher rates of decomposition and mineralization. The concentration of POC in cultivated areas is mainly affected by tillage practices [62]. A study done by [49] to compare conventional tillage and no-till showed that POC decreased in the conventional tillage and increased in the no-till management. It was hypothesized that the breakdown of macro-aggregates and diminished binding agents leads to disintegration of the soil matrix which releases the labile carbon to a free state; this in turn increases its loss substantially from soil. In a similar study, Jacinthe et al. [63] concluded that farms with minimal cover on the soil in between seasons lost higher concentrations of labile carbon compared to those with cover.

Grasslands recorded low POC levels which were not significantly different from the BL (Fig. 4). These results are similar to those obtained by [11] in the desert steppe in Mongolia, which reflected low POC concentrations in medium and high intensity grazing management systems in China. This was attributed to low surface cover, low root biomass and the vulnerability of the soil to erosion. Herbivore influence on POC in soil is also reflected on selective harvesting of above ground biomass. Li et al. [64] reported that over extraction of green succulent herbage with little input leads to low POC, while other studies suggest that controlled grazing triggers enzymes that increase microbial activities leading to mineralization in the short term [65]. In the grasslands of northern Great Plains showed that different grazing regimes influenced plant species diversity, which showed correlation with high turnover carbon. The results indicate that high grazing intensity resulted in increased competition for easily available carbon, therefore reduced labile carbon. In contrast, other researchers concluded that grazing intensity led to decrease in plant function rather than low nutrient accumulation and that the latter was primarily due to erosion [66–68].

Due to exposure of the surface by overgrazing and patches of bareland, the erosion process has influenced the lateral carbon fluxes in each land use [7, 69, 70]. Similar studies by [8, 71] illustrate that POC being the lighter fraction is easily carried away in semi-arid areas with poor soil management structures. The POC waslow in all land use types. This may be because POC is generally lower compared to MOC in soil; as it is related to light sand size fractions that are easily carried away by water erosion [72]. Furthermore, POC does not form organo-complexes with minerals therefore making it susceptible to mineralization [73]. Comparable results are seen in woodlands of Tanzania where enrichment of POC to the total was lower than that of the, stable or the silt–clay organic fractions [74].

### Mineral organic carbon (MOC)

Mineral organic carbon or stable organic carbon was not as sensitive to land uses as POC. These results are comparable to those of [75] in India and [76] in Northern China which showed that the recalcitrant material showed minimal decrease across different land use types most likely due to the inaccessibility of MOC because of the strong bonds created between the clay surfaces and the soil organic carbon. Other studies have shown that MOC is more sensitive to land use management for example [21] working in the Kenyan central Highlands showed that different potato cropping systems affect the stable fraction more than the labile one. While [77] observed in Northern Germany that MOC was more sensitive to land use change compared to TOC. This could be attributed to the fact that erosion sorts out particles mainly according to their sizes, in which in the areas have high levels of clay particles compared to the study area of this research. Therefore the finer silt and clay particles were highly enriched with MOC therefore mobilised larger quantities.

### Carbon management index (CMI)

For EN_POC_, SH registered the highest values (Table 2). This is because SH provide a less oxidative environment for POC breakdown, due to the presence of the thicket canopy, protective structure of the macroaggregates and lower erodability enabling POC build-up. These results are similar to those obtained by [4] that showed low disturbance in native grasslands increased the lability of carbon to TOC. Similarly, in Brazil, [78] undertook a study to evaluate no-till management system and compared it to a native pasture land with minimum disturbance. The results illustrated that higher EN_POC_ was recorded in the enclosed pastures similar to those with no-till of up to 20 years. The lower levels of EN_POC_, CPI and LI in GR indicate that this land use type is at a more advanced stage of degradation compared to AG which has been under cultivation for the last 7 years [36]. This translates to lower C inputs and higher turnover rates due to high temperature as well as SOC erosion. Similar results have been obtained by [11, 79].

The high CMI values in AG may be linked to the use of fertilizer on the farms. The use of nitrogen based fertilizer has been seen to increase biomass therefore increase soil organic matter in soil. These results are comparable to [14] who showed that in corn cropping systems, addition of fertilizer and stubble increases the lability of SOM by 12–46% therefore increasing CMI. In GR, overgrazing was seen to reduce the C content which can be attributable to reduction of herbaceous fine root biomass [80] thereby reducing the CMI of grasslands.

There is no definite standard for CMI as it is based on the native land use of an area; however [4] suggested that higher CMI values indicate rehabilitation of carbon while lower CMI values show that the C is being degraded. Moreover, according to [81] the land use with the higher CMI seems to provide better options for C rehabilitation.

## Conclusions

This study shows that different land use types have an influence on soil organic carbon pools and consequently the CMI. The labile fraction represented by POC is low across all the land use types and at different soil depths. Shrublands had the highest POC value which may be attributed to higher litter input and low disturbance compared to the other LUTs. The levels of POC in AG are linked to the use of fertilizer and intercropping that is practised in the catchment. In grasslands the unexpectedly lower POC levels are linked to the high levels of over grazing leading to low herbaceous litter input. The MOC was higher than the POC due to the fact that it is not easily influenced by soil management systems. In order to assess the sensitivity of the POC to LUTs, the CMI showed that level of degradation in the GR was as severe as that of BL. Therefore efforts aimed at improving SOM within each land use types will improve the soil quality and otherwise reverse degradation within the catchment. The study recommends immediate action on the grazing management strategies to reduce above ground biomass harvesting to encourage build-up of SOC. Soil management strategies should be employed in the agricultural areas to increase the labile pool consequently improve the long term fertility of the soils.
